# Ill Health from New Houses

**Published:** 1914-07

**Authors:** 


Ill Health from New Houses.—Dr. A. M. Fraser calls attention to the dangers that come from moving into new houses before they are thoroughly dried, after being finished. He recommends a certificate from public health officials to the effect that a new house will not because of its newness injure the health of its occupants. Such municipal regulations now exist in England, and are said to be proving efficacious and satisfactory. The certificate must specify that the house to be rented is sanitary in every respect and fit for occupancy. Dr. Fraser says: "I would far sooner live in a house with defective drainage than in a damp house. The results from the latter are more insidious in their onset and more difficult to overcome. Dampness undoubtedly greatly favors the incidence of consumption, bronchitis, rheumatism, heart disease and diphtheria. Probably children are more susceptible to the ill effects of damp houses than adults."

The departments of health of our cities might well take up this question. Probably most American municipalities already have the legal power necessary to require such certificates, and the question is only one of making the regulation and establishing the custom.

